# Exercise training in kidney transplant recipients (EXTRA Trial): protocol for a randomized controlled trial

**DOI:** 10.3389/fneph.2026.1972911

**Published:** 2026-10-26

**Authors:** Rafael L. Santos, Thiago S. Rosa, Samara Sezana-Costa, Suyane R. Lisboa, Marco Aurélio M. Estivalet, Carlos Ernesto Santos Ferreira, Gislane Ferreira de Melo, Lysleine Alves de Deus, José Adeirton, Michel K. Souza, Anderson S. Haro, Fernando Costa, Mariana Campos A. Mattos, José R. B. Neto, Carmen Tzanno-Martins, Daniel M Mendes, Guilherme T. Ribas, Geraldo R. R. Freitas, Mauricio Castro-Sepulveda, Adriana Bañares-Cruz, Thiago J. Borges, Leonardo V. Riella, Rodrigo P. Neves, Hugo L. Correa

**Affiliations:** 1 Department of Exercise Sciences and Physiology, Graduate Program in Physical Education, Catholic University of Brasília, Brasília, Distrito Federal, Brazil; 2 Laboratory for Science and Innovation Applied to Pathophysiology and Exercise, Catholic University of Brasília, Brasília, Distrito Federal, Brazil; 3 Department of Genomic Sciences and Biotechnology, Catholic University of Brasília, Brasília, Distrito Federal, Brazil; 4 State University of Northern Paraná, Jacarezinho, Paraná, Brazil; 5 Department of Nephrology, Federal University of São Paulo, São Paulo, Brazil; 6 Nefroclínicas Group, Belo Horizonte, Minas Gerais, Brazil; 7 Clinical Group Home Dialysis Center and RenalClass, São Paulo, Brazil; 8 Department of Surgery, Center for Transplantation Sciences, Massachusetts General Hospital, Harvard Medical School, Boston, MA, United States; 9 Luxembourg Centre for Systems Biomedicine (LCSB), University of Luxembourg, Esch-sur-Alzette, Luxembourg; 10 Division of Transplantation, University Hospital of Brasília, HUBrasil, Brasília, Distrito Federal, Brazil; 11 Center for Exercise Physiology and Metabolism, Department of Kinesiology, Faculty of Medicine, Universidad Finis Terrae, Santiago, Chile; 12 Facultad de Medicina, Universidad Autónoma de Yucatán, Mérida, Yucatan, Mexico

**Keywords:** cardiorespiratory fitness, exercise training, graft survival, immune regulation, inflammation, kidney transplantation, PD-1/PD-L1

## Abstract

Kidney transplantation improves survival and quality of life compared with long-term dialysis, yet many kidney transplant recipients remain characterized by reduced physiological reserve, cardiometabolic vulnerability, and ongoing graft-related risk. Structured exercise is a promising non-pharmacological strategy after transplantation, but its potential immunometabolic and immunomodulatory effects and their relationship with longer-term graft-related trajectories remain insufficiently characterized. The EXercise Training in Kidney TRAnsplant Recipients Trial (EXTRA Trial) is a randomized, assessor-blinded, parallel-group controlled trial that enrolled adult kidney transplant recipients approximately 6 months after transplantation. Participants are allocated 1:1 to usual post-transplant care or a supervised, progressive, combined aerobic and resistance exercise program consisting of a 2-week familiarization period followed by 24 weeks of training. The primary outcomes are the between-group differences in change in peak oxygen uptake (VO_2_peak) and handgrip strength from baseline to the end of the randomized intervention. VO_2_peak serves as the principal cardiorespiratory outcome and the basis for the sample-size calculation. Major secondary outcomes include patient-reported physical functioning, habitual physical activity, health-related quality of life, body composition, renal function, blood pressure, and metabolic markers. Exploratory outcomes include rejection-free survival, graft survival, circulating inflammatory cytokines (TNF-α, IL-6, and IL-2), soluble PD-1 and PD-L1, and longer-term trajectories assessed through 24 months after transplantation. The protocol follows SPIRIT 2025 recommendations and integrates exercise physiology, transplant medicine, and immune-related phenotyping to examine whether exercise-induced improvements in physiological reserve are accompanied by measurable inflammatory, immune-checkpoint, and graft-related changes

**Clinical trial registration:**

ClinicalTrials.gov, identifier NCT06216015.

## 1 Introduction

Kidney transplantation is the preferred treatment for patients with kidney failure, providing superior survival and quality of life compared with long-term dialysis (1–3). However, many kidney transplant recipients continue to experience reduced physiological reserve, persistent cardiometabolic vulnerability, and ongoing graft-related risk despite successful surgery and contemporary immunosuppressive therapy (1–6). Although advances in immunosuppression have substantially improved short-term graft outcomes (3, 7, 8), rejection and chronic allograft injury remain important barriers to durable transplant success (3, 6, 8, 9).

This residual risk creates a clinical window in which non-pharmacological strategies may complement standard post-transplant care (10–12). Among these strategies, structured exercise training is particularly relevant because it acts as a systemic physiological stimulus rather than a single-target intervention (13, 14). By improving cardiorespiratory fitness, skeletal muscle function, body composition, vascular health, metabolic regulation, inflammatory balance, and health-related quality of life, exercise may help rebuild physiological reserve during post-transplant recovery. In kidney transplant recipients, exercise training appears feasible and safe, and randomized trials suggest benefits for cardiorespiratory fitness, exercise capacity, muscle strength, and quality of life (5, 10, 12, 15–18). This evidence is reflected in clinical guidelines, although the specificity of their recommendations varies. The KDIGO guideline for the care of kidney transplant recipients recommends that patients be strongly encouraged to follow a healthy lifestyle that includes exercise, a recommendation based on low-quality evidence (19). The UK Kidney Association guideline is more specific: it recommends that kidney transplant recipients aim for 150 min of moderate-to-vigorous physical activity per week and suggests structured exercise combining aerobic and resistance training at least three times per week, supervised by appropriately trained staff when possible (20). However, the evidence informing these recommendations has largely framed exercise as a functional and cardiometabolic intervention. Its potential role as an immunometabolic and immunomodulatory strategy after transplantation remains insufficiently explored, particularly in relation to rejection-related events and long-term clinical trajectories.

Preclinical transplant models provide biological plausibility for this framework (21, 22). Experimental evidence suggests that voluntary exercise can modulate inflammatory (22) and regulatory immune pathways (21), thereby increasing graft survival. These findings raise the possibility that exercise may contribute to a more favorable immune-metabolic environment. Whether these adaptations can be captured in a structured clinical trial with longitudinal graft-related follow-up remains unclear. The EXTRA Trial was designed to evaluate a progressive combined aerobic and resistance exercise training program beginning approximately 6 months after kidney transplantation and ending at approximately 12 months post-transplantation. The primary randomized comparison is performed at the end of the intervention. Participants are subsequently followed for an additional 12 months, through 24 months post-transplantation, as part of an exploratory observational follow-up. This protocol describes the rationale, study design, intervention, assessment procedures, safety monitoring, outcome framework, and analytical plan of the EXTRA trial.

## 2 Methods and analysis

### 2.1 Study design and reporting framework

The study protocol is reported in accordance with the Standard Protocol Items: Recommendations for Interventional Trials (SPIRIT) 2025 statement (23). A completed SPIRIT checklist is provided as Supplementary Material 1. Prespecified statistical and artificial intelligence-based analyses will be conducted in accordance with contemporary best practices for research involving human participants (24), with prediction-model development and reporting guided by the TRIPOD+AI framework to promote transparency, reproducibility, and appropriate model evaluation (Supplementary Material 2).

The EXTRA Trial is a randomized, controlled, parallel-group clinical trial designed to evaluate the effects of a supervised exercise training program in kidney transplant recipients. Participants were enrolled approximately 6 months after kidney transplantation and allocated to either a structured exercise training group or a usual-care control group. Both groups receive standard post-transplant care throughout the study period. Participants allocated to the exercise group receive the same standard care plus a supervised program combining aerobic and resistance training. The randomized intervention period extends from approximately 6 to 12 months post-transplantation. The assessment performed at approximately 12 months post-transplantation represents the primary post-intervention assessment. After completion of the intervention, participants enter an additional 12-month observational follow-up phase, with a final exploratory assessment performed at 24 months post-transplantation to evaluate the durability of physiological adaptations and longer-term clinical trajectories. The randomized intervention and participant follow-up have been completed, whereas data analysis and scientific dissemination remain ongoing. The present manuscript provides a detailed account of the trial methods before publication of the trial findings. The overall study design is presented in **Figure 1**.

**Figure 1:**
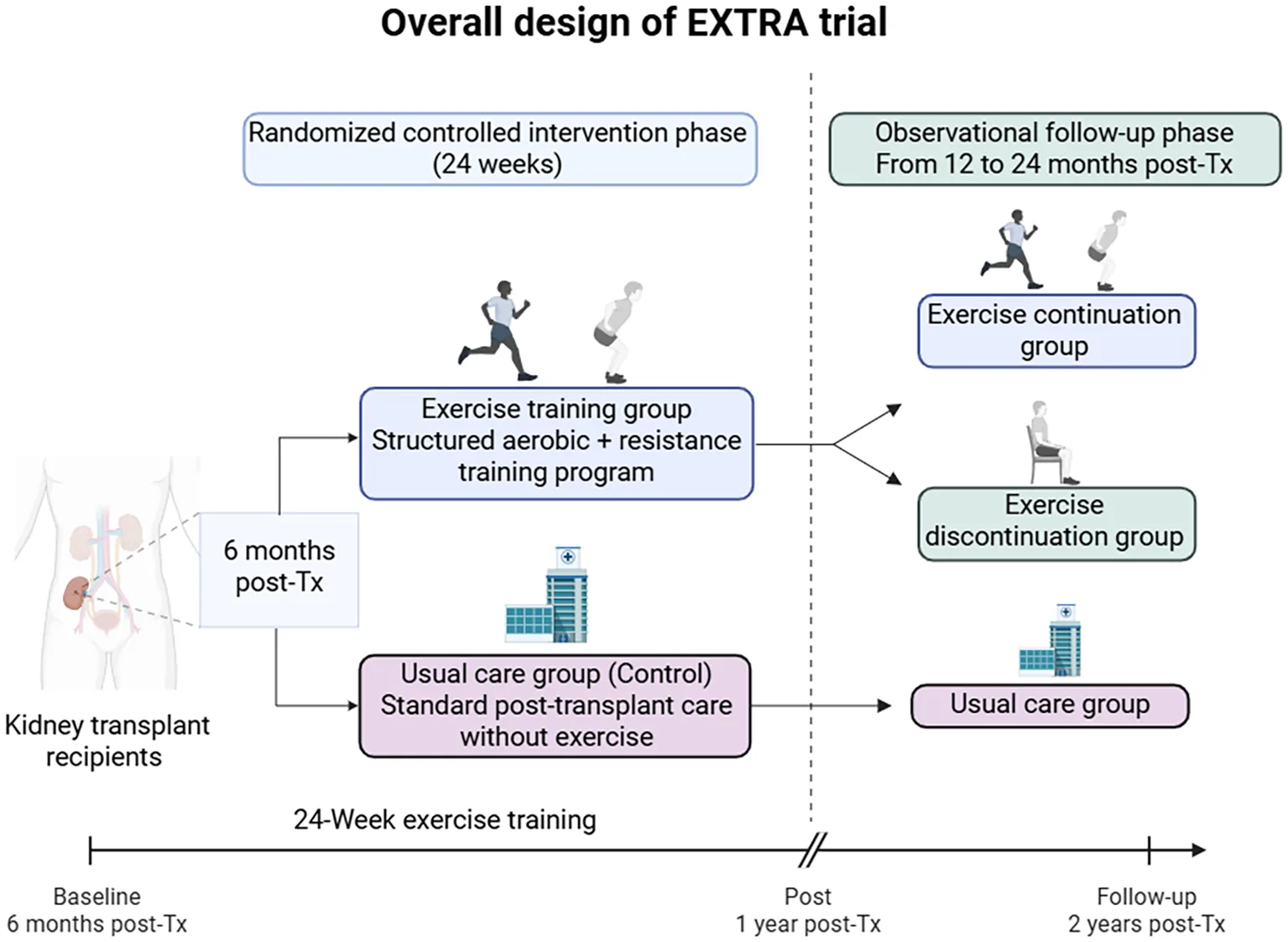
Overall design of the EXTRA Trial. Kidney transplant recipients are enrolled approximately 6 months after transplantation and randomized to either a supervised exercise training program or usual post-transplant care. The randomized controlled intervention phase lasts 24 weeks and compares structured combined aerobic and resistance training with standard post-transplant clinical follow-up without a structured exercise intervention. After completion of the randomized intervention phase, participants originally allocated to exercise enter an observational follow-up phase and are classified according to post-intervention exercise behavior as exercise continuers or exercise discontinuers. Participants allocated to usual care continue to be followed as the control group. Follow-up assessments are performed up to 24 months after transplantation. Tx, transplantation. Flow diagram of the EXTRA Trial showing kidney transplant recipients enrolled approximately 6 months after transplantation and randomized to supervised combined aerobic and resistance exercise training or usual care. The randomized intervention lasts 24 weeks, followed by an observational phase through 24 months post-transplantation in which exercise participants are classified according to exercise continuation or discontinuation.

Due to the nature of the intervention, participants and exercise professionals cannot be blinded to group allocation. However, outcome assessors, laboratory personnel, and the data analyst are blinded to group allocation, as described under randomization and allocation. The study was designed to assess physical, functional, body composition, biochemical, and clinical outcomes across the post-transplant follow-up period.

### 2.2 Trial status

Participant recruitment, the randomized intervention, and longitudinal participant follow-up have been completed. ClinicalTrials.gov currently reports a study start in December 2015, an actual enrollment of 345 participants, primary completion on November 3, 2023, and study completion on December 5, 2023. The trial record was first submitted to ClinicalTrials.gov on January 1, 2024, after initiation of the study, and therefore constitutes retrospective registration. No trial outcome data or between-group comparative results are reported in the present manuscript. Preparation and dissemination of the trial outcome reports remain ongoing. The present article provides a consolidated methodological description of the EXTRA Trial before publication of its outcome reports.

### 2.3 Study setting, ethics approval, and trial registration

The EXTRA Trial is a single-center randomized controlled trial conducted at the Catholic University of Brasília, Brazil, with participant referral and recruitment support from collaborating kidney transplant services. Intervention delivery, physiological assessments, and study coordination are centralized at the study clinic. The exercise intervention is delivered in person using a small-group model, typically involving approximately 4–5 participants per session. Exercise sessions and physiological assessments are conducted in supervised facilities equipped for exercise testing, resistance training, body-composition assessment, biological sample collection, and clinical monitoring. Participants allocated to usual care continue to receive standard post-transplant clinical follow-up in accordance with institutional routines and the recommendations of the transplant care team.

The original kidney-transplant exercise project was approved by the Research Ethics Committee of the Federal University of São Paulo under CAAE 50129315.2.0000.5505. A separate comprehensive research program at the Catholic University of Brasília, which includes kidney transplant recipients, was subsequently approved under CAAE 79266224.1.0000.0029.

### 2.4 Participants

Eligible participants are adult kidney transplant recipients enrolled approximately 6 months after transplantation. Participants are screened for clinical stability and ability to perform physical assessments and exercise training. Eligibility for physical assessments and exercise training is determined through a pre-participation clinical evaluation, including the ability to ambulate independently, follow exercise instructions, and safely complete the baseline physical assessment battery. Participants with acute clinical conditions or contraindications to exercise, as determined by the treating physician, are not eligible to enroll. Exclusion criteria include clinical contraindications to exercise testing or training, acute medical instability, active infection, recent cardiovascular events, severe musculoskeletal limitations preventing exercise participation, or any clinical condition judged by the study team to compromise participant safety.

All participants provide written informed consent before any study procedure. Demographic information, clinical history, donor type, transplant-related variables, medication use, and comorbidity profile are collected at baseline.

### 2.5 Sample size calculation

The sample-size calculation was based on VO_2_peak, one of the two registered primary outcomes, because it provides an integrative measure of cardiorespiratory and whole-body physiological capacity and because suitable effect-size estimates were available from previous randomized exercise trials in kidney transplant recipients. Previous evidence in kidney transplant recipients suggests that supervised exercise training may improve VO_2_peak by approximately 2.4 mL·kg^-^¹·min^-^¹. Assuming a between-group difference of 2.42 mL·kg^-^¹·min^-^¹, a common standard deviation of 6.0 mL·kg^-^¹·min^-^¹, a two-sided alpha level of 0.05, and 80% statistical power, 97 participants per group were required. The assumed difference of 2.42 mL·kg^-^¹·min^-^¹ is the pooled between-group mean difference (exercise versus control; 95% CI, 0.22 to 4.63 mL·kg^-^¹·min^-^¹) reported in a meta-analysis of randomized trials of supervised exercise training in kidney transplant recipients (25). It therefore represents a between-group effect rather than a within-group change, and it is smaller than the pooled effect of 3.87 mL·kg^-^¹·min^-^¹ reported in a more recent meta-analysis (15). The common standard deviation of 6.0 mL·kg^-^¹·min^-^¹ was set conservatively, above the between-patient variability described in pooled data from kidney transplant recipients (VO_2_peak of 21.6 ± 4.9 mL·kg^-^¹·min^-^¹ beyond 12 months after transplantation) (5). No minimal clinically important difference in VO_2_peak has been established for kidney transplant recipients. The target difference corresponds to approximately 0.7 metabolic equivalents (MET) and to about 11% of the mean VO_2_peak reported in this population (5), a magnitude with prognostic relevance in other populations: each 1-MET higher level of cardiorespiratory fitness was associated with 13% lower all-cause mortality in a meta-analysis of cohort studies (26), and in chronic heart failure each 6% increase in peak VO_2_ was associated with 7% lower all-cause mortality (27). In kidney transplant candidates, lower pre-transplant VO_2_peak has also been associated with higher post-transplant mortality (28). To account for an anticipated attrition rate of 20%, the sample size was inflated to 121 participants per group, yielding an estimated sample size of 242 participants for the VO_2_peak randomized comparison. This value represents the statistical sample-size estimate and was not a recruitment ceiling; the actual enrollment was 345 participants, as reported in ClinicalTrials.gov.

Given that rejection-related events are expected to occur less frequently than changes in continuous physiological outcomes, analyses of rejection-free survival and other graft-related events will be interpreted based on the observed number of events, confidence intervals, and clinical relevance.

### 2.6 Randomization and allocation

After completion of baseline assessments, eligible participants are randomized in a 1:1 allocation ratio to either supervised exercise training or usual care. The allocation sequence was computer-generated by an independent researcher who is not involved in recruitment, eligibility assessment, intervention delivery, outcome assessment, or data analysis. This researcher prepared sequentially numbered, sealed, opaque envelopes containing the group assignments and keeps the allocation list in a secure location that the rest of the study team cannot access. After a participant has completed all baseline assessments, a member of the study team who does not perform outcome assessments opens the next envelope in consecutive order and records the envelope number, date, and allocation. Participants allocated to the exercise group complete a 2-week familiarization period followed by a 24-week structured, progressive, combined aerobic and resistance training program. Participants allocated to the usual care group continue standard post-transplant clinical follow-up without a structured exercise intervention during the randomized phase. Participant enrollment, allocation procedures, and intervention coordination are conducted by study personnel. Because of the nature of the intervention, participants and exercise professionals cannot be blinded to group allocation; however, outcome assessors, laboratory personnel, and the data analyst are blinded. Cardiopulmonary exercise testing, dual-energy X-ray absorptiometry, handgrip strength, blood pressure, and questionnaire administration (IPAQ and SF-36) are performed by assessors who do not deliver the intervention and are not informed of group allocation, and participants are asked not to disclose their allocation or discuss their training during assessment visits. Blood and urine samples are labeled with coded identifiers, so that clinical biochemistry and enzyme-linked immunosorbent assays are performed without knowledge of allocation. The data analyst receives a dataset in which the randomized groups are coded (A and B), and the code is revealed only after the primary analysis has been completed. Rejection-related and graft-related events are adjudicated by investigators unaware of group allocation. Blinding is maintained throughout the assessment and analysis periods, and any instance of assessor unblinding is recorded and reported. The allocation structure and transition from the randomized intervention phase to the observational follow-up phase are summarized in **Figure 1**.

### 2.7 Assessment procedures

Participant assessments are conducted using standardized operating procedures to ensure reproducibility across time points. The full assessment workflow is shown in **Figure 2**. Anthropometric measurements include body weight and height, assessed using a calibrated Filizola scale and stadiometer. Habitual physical activity is assessed in participants from both the exercise and usual-care groups using the International Physical Activity Questionnaire (IPAQ) at baseline, at the end of the randomized intervention, and at the 24-month post-transplant follow-up. The short form of the IPAQ (last 7-day recall) is used in its version validated for Brazilian Portuguese (29, 30) and is administered by trained assessors. Resting systolic and diastolic blood pressure are measured using an oscillometric device. Body composition is assessed by dual-energy X-ray absorptiometry, and muscle strength is assessed by handgrip dynamometry using a Jamar dynamometer. Blood samples are collected for renal function markers, fasting blood glucose, lipid profile, C-reactive protein, inflammatory cytokines, and immune-checkpoint markers. Lipid profile is measured on a Cobas clinical chemistry platform, whereas cytokines, PD-1, and PD-L1 are measured by enzyme-linked immunosorbent assay. Spot urine and 24-hour urine collections are obtained for analysis of the urinary and renal systems. Rejection-related events and graft-related outcomes are identified from clinical records and transplant follow-up documentation. Detailed procedures, instruments, operational definitions, and quality-control procedures for each outcome domain are provided in Supplementary Material 3.

**Figure 2:**
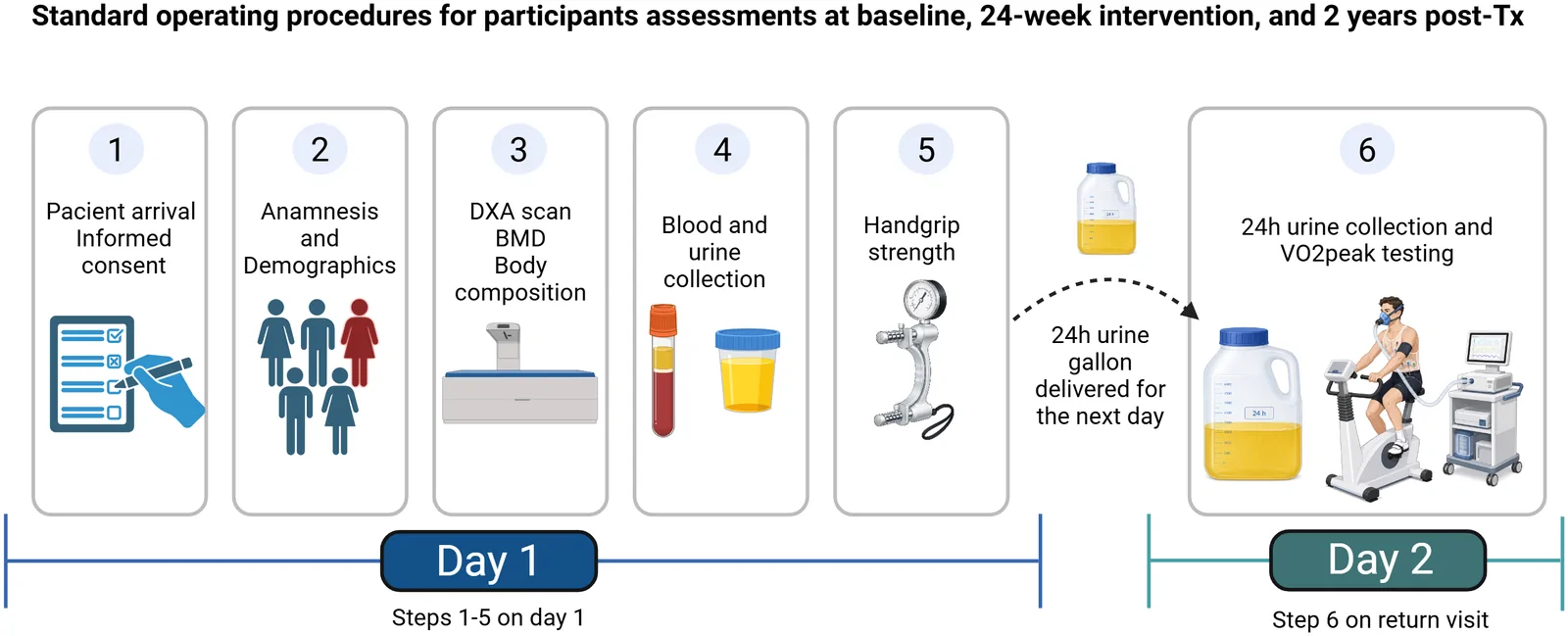
Standard operating procedures for participant assessments. Participant assessments are performed using standardized operating procedures at baseline, after the 24-week intervention, and during long-term follow-up up to 24 months after transplantation. On the first assessment day, participants complete informed consent procedures, anamnesis and demographic characterization, dual-energy X-ray absorptiometry for bone mineral density and body composition, blood and spot urine collection, and handgrip strength testing. Participants receive instructions and materials for 24-hour urine collection. On the return visit, participants deliver the 24-hour urine sample and undergo cardiopulmonary exercise testing for determination of VO_2_peak. BMD, bone mineral density; DXA, dual-energy X-ray absorptiometry; Tx, transplantation; VO_2_peak, peak oxygen uptake. Diagram of the standardized assessment workflow used in the EXTRA Trial. Assessments include clinical and demographic characterization, dual-energy X-ray absorptiometry, blood and urine collection, handgrip strength, and cardiopulmonary exercise testing for VO_2_peak, performed at baseline, post-intervention, and long-term follow-up.

### 2.8 Exercise intervention

Participants randomized to the exercise group complete an in-person, small-group supervised exercise training program delivered at the study clinic, typically with approximately 4–5 participants per session. Each session is supervised by two exercise professionals, both trained in basic life support and in the study protocol, which gives a ratio of approximately one professional to two or three participants. Although sessions are conducted in groups, exercise progression and external loads are individually adjusted according to participant tolerance, clinical status, perceived exertion, and safety criteria. The randomized intervention phase begins approximately 6 months after kidney transplantation and ends at approximately 12 months post-transplantation. Participants are subsequently followed for an additional 12 months, through 24 months post-transplantation. Participants randomized to the exercise group complete a supervised progressive exercise training program comprising a 2-week familiarization period followed by a 24-week combined resistance and walking-based aerobic exercise intervention. Therefore, the structured training phase lasts 24 weeks; including the preceding 2-week familiarization period, the post-training assessment occurs approximately 26 weeks after randomization. During the familiarization period, participants are introduced to the exercise equipment, movement patterns, session procedures, and safety instructions before commencing the progressive training phases. The intervention is organized into four phases. The first three phases last 4 weeks each and progressively increase training volume and exercise complexity. From weeks 13 to 24, participants maintain the phase 3 exercise structure, with progressive load increases as tolerated. The exercise training model is summarized in **Figure 3**.

**Figure 3:**
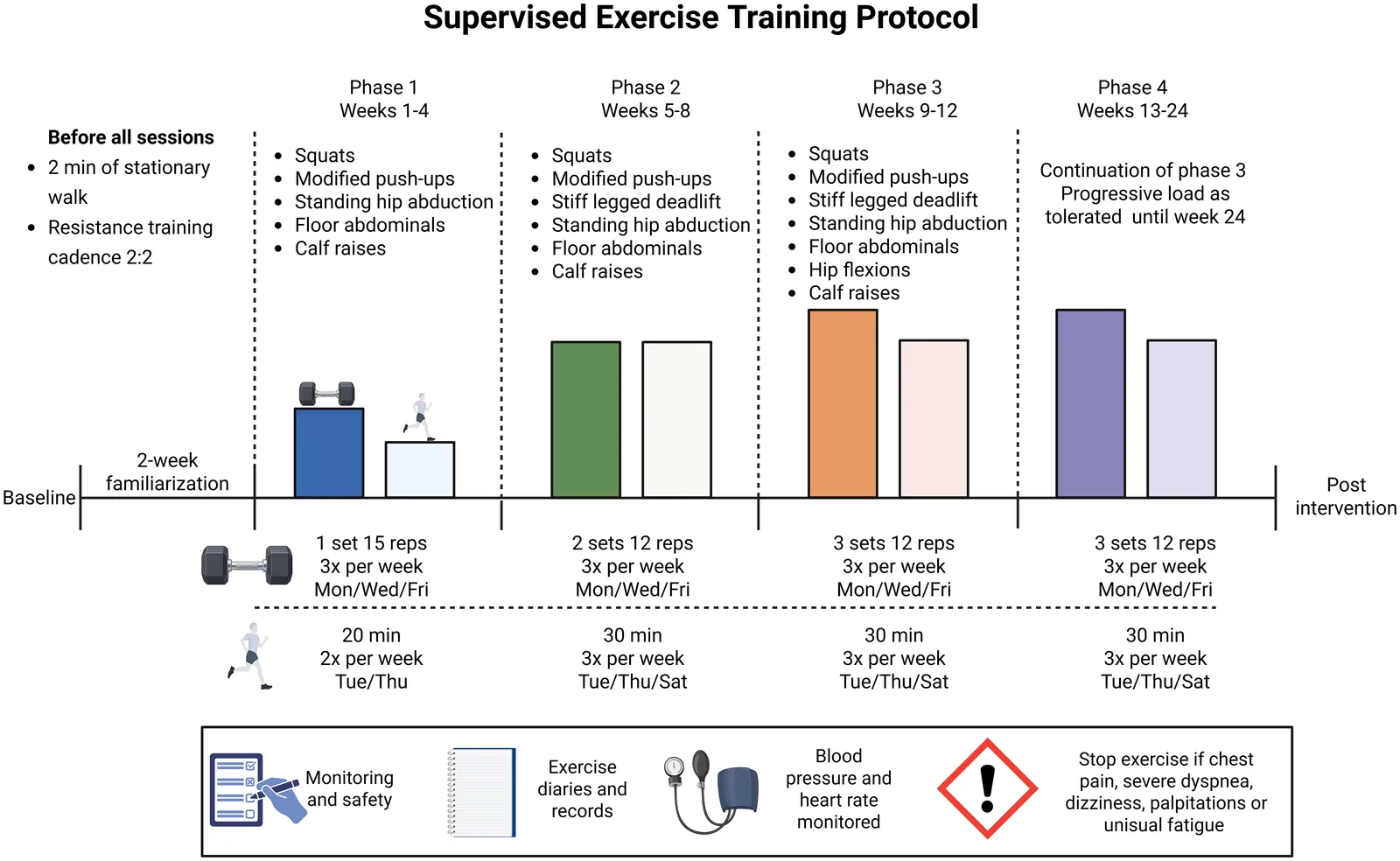
Supervised exercise training protocol. All sessions are conducted in person at the study clinic using a small-group delivery model, typically involving approximately 4–5 participants per session, with individualized exercise progression and safety monitoring. The exercise intervention consists of a 2-week familiarization period followed by a 24-week supervised, progressive, combined aerobic and resistance training program. Before all sessions, participants perform 2 minutes of stationary walking as a warm-up, and resistance exercises are performed using a 2:2 cadence (i.e., 2 seconds during the concentric phase and 2 seconds during the eccentric phase). After familiarization, the protocol progresses through four phases. In phase 1, participants perform 1 set of 15 repetitions of resistance exercises three times per week and 20 minutes of walking twice per week. In phase 2, resistance training progresses to 2 sets of 12 repetitions and aerobic walking to 30 minutes per session. In phase 3, resistance training progresses to 3 sets of 12 repetitions and aerobic walking to 30 minutes per session. During phase 4, from weeks 13 to 24, walking is maintained at 30 min/session, three times per week, with progression as tolerated. Exercise adherence, perceived exertion, blood pressure, heart rate, symptoms, and adverse events are monitored throughout the intervention. Schematic of the 24-week supervised combined exercise intervention following a 2-week familiarization period. Resistance training progresses from 1 set of 15 repetitions to 3 sets of 12 repetitions, while aerobic walking progresses from 20 to 30 minutes per session. From weeks 13 to 24, training volume is maintained with individualized progressive increases in external load.

Before all training sessions, participants perform 2 minutes of stationary walking as a warm-up. Resistance training is performed three times per week on Monday, Wednesday, and Friday. In phase 1, participants perform 1 set of 15 repetitions of squats, modified push-ups with knee support, standing hip abduction, floor abdominal exercises, and calf raises. In phase 2, resistance training progresses to 2 sets of 12 repetitions and includes squats, modified push-ups, stiff-legged deadlifts, standing hip abduction, floor abdominal exercises, and calf raises. In phase 3, resistance training progresses to 3 sets of 12 repetitions and includes squats, modified push-ups, stiff-legged deadlifts, standing hip abduction, abdominal exercises, hip flexion, and calf raises. During phase 4, from weeks 13 to 24, the phase 3 structure is maintained, with progressive load increases according to individual tolerance and safety criteria.

Aerobic training consists of walking sessions performed on non-resistance training days. In phase 1, participants walk for 20 minutes twice per week (Tuesday, Thursday). In phase 2, walking progresses to 30 minutes per session three times per week (Tuesday, Thursday, and Saturday). In phase 3, walking is maintained at 30 minutes per session three times per week (Tuesday, Thursday, and Saturday). During weeks 13 to 24, aerobic training is maintained the same as phase 3, with progression as tolerated. Exercise frequency, weekly structure, resistance training volume, aerobic duration, and safety monitoring procedures are detailed in **Figure 3**. All resistance and aerobic exercise sessions are delivered in person at the study facilities and are directly supervised by qualified exercise professionals. Attendance, perceived exertion, symptoms, heart rate, and blood pressure are monitored according to the participant’s clinical status and prespecified safety procedures.

### 2.9 Monitoring and safety

Safety procedures are implemented throughout the intervention. Before each session, participants are asked about symptoms, intercurrent illness, hospital admissions, and medication changes since the previous session, and resting blood pressure and heart rate are measured after a period of seated rest. The session is postponed if any of the following is present: resting systolic blood pressure ≥180 mmHg or diastolic blood pressure ≥110 mmHg; systolic blood pressure <90 mmHg with symptoms; resting heart rate >100 bpm, or <50 bpm with symptoms; a new irregular rhythm or palpitations; body temperature ≥37.8 °C or symptoms of acute infection; or, in participants with diabetes, capillary glucose <70 mg/dL or >300 mg/dL. These criteria were adapted from the American Heart Association exercise standards (31).

Participants are instructed to stop exercise and contact the study team if they experience chest pain, severe dyspnea, dizziness, palpitations, unusual fatigue, or any other concerning symptom. During supervised sessions, blood pressure and heart rate are monitored based on clinical judgment and the participant’s risk profile. Exercise is stopped immediately in case of chest pain or angina-like symptoms, severe dyspnea, dizziness or presyncope, confusion, pallor or cyanosis, palpitations or a perceived irregular heartbeat, unusual fatigue, musculoskeletal pain, systolic blood pressure >250 mmHg or diastolic blood pressure >115 mmHg, or a fall in systolic blood pressure >10 mmHg despite increasing effort (31). Participation is suspended, and resumed only after clearance by the transplant team, in case of hospitalization, acute infection requiring treatment, suspected or confirmed rejection, acute graft dysfunction under investigation, surgical or graft-site complications, or a new cardiovascular event. The intervention is permanently discontinued in case of graft failure, a serious adverse event related to exercise that contraindicates further training, withdrawal of consent, or a decision of the treating physician; participants who stop the intervention remain in follow-up and are analyzed in their allocated group.

Adverse events are defined as any untoward medical occurrence in a participant, whether or not it is considered related to the intervention, and serious adverse events as those that result in death, are life-threatening, require hospitalization or prolong an existing hospitalization, result in persistent or significant disability, or are considered important medical events. Adverse events are recorded actively at every supervised session in the exercise group and at every assessment visit and scheduled follow-up contact in both groups, and this information is complemented by review of medical records. Serious adverse events are reported to the principal investigator within 24 hours and notified to the research ethics committee in accordance with national regulations. Operational definitions are detailed in Supplementary Material 3.

Exercise adherence is monitored using exercise diaries, attendance records, and weekly perceived exertion was assessed using the Brazilian Portuguese version of the Borg 6–20 Rating of Perceived Exertion scale (32). These records are reviewed during follow-up contacts to assess attendance, perceived exertion, difficulties with exercise execution, and the need for progression or adjustment. Participants are instructed to avoid exercise during acute illness and to follow individualized clinical guidance regarding hydration and fluid intake. Adherence is calculated as completed sessions divided by prescribed sessions × 100 over the 24-week intervention, excluding familiarization (140 prescribed sessions: 72 resistance and 68 aerobic). Missed sessions count as not completed and their reasons are recorded; sessions postponed or suspended for clinical reasons remain in the denominator; partially completed sessions contribute the proportion of the prescribed volume performed (sets and repetitions, or walking minutes). Participants completing at least 70% of prescribed sessions are classified as adherent.

### 2.10 Usual care

Participants allocated to the usual care group receive standard post-transplant clinical care without a structured exercise training program during the randomized intervention phase. Usual care includes routine medical follow-up, medication management, laboratory monitoring, and clinical recommendations provided by the transplant care team. In practice, this comprises scheduled outpatient visits with the transplant team, monitoring and adjustment of immunosuppressive therapy, laboratory monitoring of graft function, and management of comorbidities such as hypertension, diabetes, and dyslipidemia. Within this routine, transplant teams give general verbal advice to remain physically active, but no individualized exercise prescription, supervised training, or structured follow-up of exercise is provided. Participants in the usual care group are not prevented from exercising on their own, and habitual physical activity is monitored with the IPAQ in both groups to quantify possible contamination.

### 2.11 Observational follow-up

After the intervention, participants in the exercise group enter an observational follow-up phase. During this phase, participants are classified according to their post-intervention exercise behavior as exercise continuers or exercise discontinuers. Exercise behavior during this phase is recorded at scheduled follow-up contacts and at the 24-month assessment. Participants who report structured aerobic and/or resistance exercise at least twice per week in at least 6 of the 12 months of the observational phase, and who accumulate at least 600 MET-min/week on the IPAQ at 24 months, are classified as exercise continuers; the others are classified as exercise discontinuers. This classification is completed before the 24-month outcomes are analyzed. Participants in the usual care arm continue to be followed as the control group. Long-term assessments are performed up to 24 months after transplantation, as illustrated in **Figure 1**.

### 2.12 Outcomes

The EXTRA Trial has two registered primary outcomes: (1) the between-group difference in change in peak oxygen uptake (VO_2_peak) from baseline to the end of the randomized intervention, measured by cardiopulmonary exercise testing; and (2) the between-group difference in change in handgrip strength over the same period, assessed by standardized handgrip dynamometry. VO_2_peak represents the primary cardiorespiratory outcome and was used for the sample-size calculation because it integrates cardiovascular, respiratory, muscular, and metabolic function. Handgrip strength represents the primary muscular-function outcome and provides a complementary assessment of peripheral muscle function. VO_2_peak was selected because it reflects the integrated function of the cardiorespiratory, muscular, and metabolic systems (33–35). Cardiopulmonary exercise testing completion, reasons for test non-completion, test-limiting symptoms, and adherence to established test-quality criteria will be reported. VO_2_peak findings will be interpreted alongside patient-reported physical functioning and other functional outcomes, recognizing the heterogeneity in baseline functional capacity and comorbidity burden among kidney transplant recipients. The SF-36 quality of life questionnaire will be considered a major patient-centered secondary outcome. Rejection-free survival, graft survival, circulating inflammatory cytokines, soluble PD-1 and PD-L1, will be analyzed as exploratory outcomes.

Body composition and bone mineral density will be assessed by dual-energy X-ray absorptiometry (36, 37), muscle strength assessed by handgrip dynamometry (38), and blood pressure assessed using an oscillometric device in accordance with American Heart Association standards (39). Renal and urinary outcomes include serum creatinine, creatinine-based estimated glomerular filtration rate, and albuminuria as markers of graft function and kidney injury (2, 19). Metabolic and biochemical outcomes include fasting blood glucose, lipid profile, and C-reactive protein (40). Immune-related exploratory outcomes include circulating inflammatory cytokines (TNF-α, IL-6, IL10, and IL-2) and immune-checkpoint markers (soluble PD-1 and PD-L1), measured by enzyme-linked immunosorbent assay according to the manufacturers’ instructions (40, 41). Health-related quality of life is assessed using the version 1 of the 36-Item Short Form Health Survey Brazilian Portuguese Version (42, 43), the eight domains (physical functioning, role limitations due to physical problems, bodily pain, general health, vitality, social functioning, role limitations due to emotional problems, and mental health) are each scored from 0 to 100, with higher scores indicating better health, and all eight will be included in the statistical analysis. Adherence is quantified as the percentage of prescribed sessions completed, as defined under Monitoring and safety, using attendance records, exercise diaries, and completion of prescribed exercise sessions. Safety is assessed through clinical monitoring, symptom reporting, adverse events, and serious adverse events throughout the intervention and follow-up (44, 45). Detailed procedures, instruments, operational definitions, and quality-control procedures for each outcome are provided in Supplementary Material 3.

The schedule of enrollment, interventions, and assessments is presented in **Table 1**. Participants are screened and enrolled approximately 6 months after kidney transplantation. After baseline assessments and randomization, participants allocated to the exercise group complete a 24-week supervised progressive exercise training program, while participants allocated to usual care continue standard post-transplant follow-up. Major assessments are performed at baseline, at the end of the 24-week intervention, and during long-term follow-up up to 24 months after transplantation. Safety, adherence, perceived exertion, adverse events, rejection-related events, and graft-related outcomes are monitored throughout the study period.

**Table 1:** Participant timeline: schedule of enrollment, interventions, and assessments following the SPIRIT checklist (23).

Procedures	Trial period						
	Enrollment		Post-randomization				Close-out
Weeks	*-2 to 0*	0	*Weeks 1-4*	*Weeks 5-8*	*Weeks 9-12*	*Weeks 13-24*	*24 months post-Tx*
Post-transplant timepoints	*6 months*	*6 months*	*6–7 months*	*7–8 months*	*8–9 months*	*9–12 months*	*24 months post-Tx*
ENROLLMENT:							
Medical clearance	X						
Eligibility screen	X						
Informed consent	X						
*Demographics and clinical history*	X						
*Donor type*	X						
*Immunosuppressive regimen and medications*	X						
Randomization		X					
INTERVENTION/ COMPARATOR:							
*Exercise Training group*		X	X	X	X	X	X
*Usual Care group*		X	X	X	X	X	X
*Exercise continuation*							X
*Exercise discontinuation*							X
ASSESSMENTS:							
*Quality-of-life questionnaires*		X				X	X
*Habitual physical activity*		X				X	X
*Dual-energy X-ray absorptiometry*		X				X	X
*Blood samples*		X				X	X
*Handgrip strength*		X				X	X
*Spot urine sample*		X				X	X
*24-hour urine collection*		X				X	X
*Cardiopulmonary exercise testing / VO_2_peak*		X				X	X
*Renal function markers*		X				X	X
*Rejection events*		X	X	X	X	X	X
*Adverse events*		X	X	X	X	X	X
*Exercise adherence*		X	X	X	X	X	X
*Borg perceived exertion scale*		X	X	X	X	X	X
*Exercise diary / attendance records*		X	X	X	X	X	X

*Training weeks 1–24 refer to the structured exercise-training period and are preceded by the 2-week familiarization period; therefore, the post-intervention assessment occurs approximately 26 weeks after randomization. Abbreviations: Tx, transplantation; VO_2_peak, peak oxygen uptake.

### 2.13 Statistical analysis

Analyses will follow the intention-to-treat principle, including all randomized participants according to their original allocation regardless of adherence, protocol deviations, or post-intervention exercise behavior. Per-protocol analyses, restricted to exercise-group participants with at least 70% adherence and to usual-care participants without major protocol deviations, will be conducted as sensitivity analyses. Descriptive statistics will summarize demographic, clinical, transplant-related, physiological, biochemical, and quality-of-life characteristics. Continuous variables will be presented as mean and standard deviation or median and interquartile range, as appropriate, whereas categorical variables will be presented as absolute and relative frequencies. Baseline characteristics will be reported descriptively, with emphasis on clinical relevance rather than formal hypothesis testing of randomization balance. The primary analyses will evaluate the between-group differences in change in VO_2_peak and handgrip strength from baseline to the end of the randomized intervention. Both outcomes will be analyzed using linear mixed-effects models including randomized group, study visit, and the group-by-visit interaction as fixed effects and a random intercept for participants. Results for both registered primary outcomes will be reported as estimated marginal means, between-group differences in change, 95% confidence intervals, and standardized effect sizes. To preserve the family-wise type I error rate across the two primary outcomes, a fixed-sequence procedure will be applied: VO_2_peak will be tested first at a two-sided α of 0.05, and handgrip strength will be tested at α = 0.05 only if the VO_2_peak comparison is statistically significant. The primary analysis will use linear mixed-effects models, including randomized group, study visit, and the group-by-visit interaction as fixed effects, and a random intercept for participants. The group-by-visit interaction at the end of the intervention will represent the primary treatment effect. Results will be reported as estimated marginal means, between-group differences in change, 95% confidence intervals, and standardized effect sizes. Beta-blocker use will be summarized by group at each visit. In a sensitivity analysis, the primary model will be refitted with beta-blocker use as a covariate, and a group-by-visit-by-beta-blocker interaction will be explored; participants whose beta-blocker therapy changes between baseline and the end of the intervention will be excluded in an additional sensitivity analysis Secondary continuous outcomes, including body composition, bone mineral density, muscle strength, blood pressure, renal function, urinary parameters, metabolic markers, inflammatory biomarkers, immune-related markers, and health-related quality-of-life scores, will be analyzed using analogous mixed-effects models when repeated measurements are available. The eight SF-36 domains will be analyzed in this way. IPAQ data will be analyzed as MET-min/week, log-transformed if necessary, and the proportion of physically active participants will be compared between groups at each visit using chi-square or Fisher’s exact tests. Baseline physical activity status (physically active or insufficiently active) will be added as a covariate in a sensitivity analysis of the primary outcome and examined as a potential effect modifier through a group-by-visit-by-activity-status interaction Long-term analyses through 24 months after transplantation will estimate trajectories based on the original randomized group allocation.

Comparisons between exercise continuers and discontinuers after the supervised intervention will be considered observational and exploratory. Missing data patterns will be described, and mixed-effects models will use all available observations under a missing-at-random assumption; multiple-imputation or complete-case sensitivity analyses may be performed when appropriate. Exploratory clinical outcomes will include rejection-free survival and graft survival. Rejection-free survival will be defined as the time from randomization to the first documented rejection-related event. Kaplan–Meier curves, log-rank tests, and Cox proportional hazards models will be used as appropriate, provided that the number of events permits stable estimation.

Binary outcomes will be analyzed using chi-square or Fisher’s exact tests, modified Poisson regression, or logistic regression, as appropriate. Exploratory responder and predictive modeling analyses will investigate interindividual variability in exercise response and baseline characteristics associated with favorable physiological, clinical, and graft-related trajectories. Candidate predictors may include demographic, transplant-related, clinical, functional, biochemical, inflammatory, immune-related, and quality-of-life variables. Candidate algorithms may include penalized regression, random forests, gradient boosting, and support vector machines. To minimize overfitting and data leakage, preprocessing, imputation, feature selection, class-balancing procedures, and hyperparameter tuning will be performed within resampling procedures. Internal validation will use repeated nested cross-validation and, where appropriate, bootstrap resampling; a fixed training–testing split will not be used as the sole validation strategy. Model performance will be assessed using outcome-appropriate measures of discrimination, calibration, error, and predictive accuracy. Feature importance and Shapley additive explanation methods may be used to support biological interpretation, but will not be interpreted as evidence of causal effects. Predictive models will require external validation before clinical application.

Statistical analyses and graphical visualizations will be performed using GraphPad Prism version 11.0.2, R version 4.6.0 implemented through RStudio IDE version 2026.06.0, and Python version 3.14.6. Mixed-effects models, survival analyses, and publication-quality graphics will be conducted primarily in R using packages including the tidyverse, lme4, lmerTest, emmeans, survival, survminer, mice, and ggplot2. Machine-learning analyses will be implemented in Python using pandas, numpy, scikit-learn, imbalanced-learn, xgboost, and shap, as appropriate. All tests will be two-sided, with statistical significance set at p < 0.05 for the primary outcomes. Analysis scripts, data dictionaries, package versions, random seeds, and reproducible workflows will be version-controlled and archived in Zenodo at publication. Each archived release will receive a persistent digital object identifier (DOI), subject to ethical approval, participant confidentiality, and applicable data-sharing restrictions.

## 3 Discussion

The EXTRA trial is designed to determine whether exercise training improves physiological reserve, with VO_2_peak and handgrip strength as the primary outcomes, while exploring associated functional, biochemical, quality-of-life, immune-related, and graft-related trajectories during post-transplant recovery. Kidney transplantation improves survival and quality of life compared with dialysis, but it does not fully restore physiological reserve or eliminate the recipient’s clinical vulnerability (1–5). Persistent deconditioning, cardiometabolic risk, chronic exposure to immunosuppression, and ongoing graft-related uncertainty create a window in which non-pharmacological strategies may meaningfully complement conventional transplant care (3, 8, 9). Within this context, the EXTRA trial integrates exercise physiology, transplant medicine, and immune-metabolic phenotyping in a randomized framework.

A key feature of the trial is the timing of the intervention. Participants are enrolled approximately 6 months after kidney transplantation, a period in which many recipients have recovered from the immediate surgical phase but remain within an early window of clinical stabilization and functional rebuilding (5). This timing is relevant because the post-transplant period is characterized by competing biological processes: recovery from kidney failure, adaptation to immunosuppressive therapy, persistence of cardiometabolic risk, and gradual restoration of physical capacity (3, 5, 8). Implementing exercise during this phase may provide a structured opportunity to improve physiological reserve before long-term patterns of inactivity, including weight gain, muscle dysfunction, and metabolic deterioration become established.

The choice of combined aerobic and resistance training reflects the nature of post-transplant impairments. Aerobic exercise targets cardiorespiratory fitness, vascular function, metabolic control, and exercise tolerance, whereas resistance training is essential for addressing muscle weakness, reduced lean mass, and functional limitations (14). This combined strategy is relevant in kidney transplant recipients, who may experience persistent sarcopenic vulnerability despite improved renal function (5). The progressive structure of the intervention, with staged increases in volume and complexity over the first 12 weeks, followed by continued progression from weeks 13 to 24, was designed to balance physiological overload with clinical safety.

VO_2_peak was selected as the key continuous physiological outcome because it represents one of the most integrative measures of human physiological reserve (33). The concept of maximal oxygen uptake emerged from the classical work of Hill and Lupton (46, 47), who proposed that, during progressively intense exercise, oxygen consumption reaches an upper limit beyond which additional external work no longer increases oxygen uptake. Since then, VO_2_max and VO_2_peak have become central measures in exercise physiology and clinical medicine, reflecting the coordinated capacity of the cardiovascular, pulmonary, vascular, hematologic, skeletal muscle, and mitochondrial systems to deliver and use oxygen during physical stress (33). From a physiological perspective, oxygen uptake is determined by the interaction between convective oxygen delivery and peripheral oxygen extraction, making VO_2_peak a whole-body marker rather than an isolated cardiopulmonary variable (34). Recent studies indicate that VO_2_peak declines across the kidney disease continuum and only partially recovers after kidney transplantation, reinforcing the notion that restoration of kidney function does not necessarily normalize cardiorespiratory fitness (5). In this context, VO_2_peak may capture dimensions of recovery that are not fully reflected by isolated biochemical markers, estimated glomerular filtration rate, or resting clinical measures (28).

The prognostic relevance of VO_2_peak is well established across general and clinical populations (48). In advanced heart failure and cardiac transplantation evaluation, peak oxygen uptake has historically been used to guide transplant candidacy and risk stratification. The landmark study by Mancini and colleagues showed that ambulatory patients with severe left ventricular dysfunction and peak VO_2_ greater than 14 mL·kg^-^¹·min^-^¹ had survival comparable to post-transplant survival, whereas lower values identified patients at substantially higher risk (49). Subsequent work by Kato and colleagues refined this framework by showing that peak VO_2_, particularly when combined with B-type natriuretic peptide, independently stratified the risk of death, heart transplantation, or ventricular assist device requirement in patients undergoing transplant evaluation (50). Kidney-specific evidence also supports the clinical relevance of this marker. In a cohort of kidney transplant recipients with at least 3 years of follow-up, lower pre-transplant VO_2_peak was associated with higher post-transplant mortality (28). Among patients classified as low risk by estimated post-transplant survival, those with low VO_2_peak showed substantially higher mortality risk, suggesting that cardiorespiratory function may reveal vulnerability not captured by conventional allocation metrics. Therefore, in the EXTRA trial, cardiopulmonary exercise testing is included to allow exploratory evaluation of cardiorespiratory fitness as a modifiable marker of post-transplant resilience and graft-related risk.

Another distinctive aspect of the EXTRA trial is the inclusion of inflammatory profile and immune-checkpoint markers as exploratory translational outcomes. Kidney allograft injury and rejection involve complex interactions between alloimmune activation, inflammatory signaling, tissue injury, and regulatory immune pathways (9). In parallel, exercise training is increasingly recognized as a systemic stimulus capable of modulating PD-L1 and regulatory T cells in pre-clinical transplant models (21, 22). Therefore, evaluating circulating cytokines, along with PD-1 and PD-L1, provides an exploratory framework to examine whether measurable immune-related changes accompany exercise-induced physiological adaptations. This is a key feature, as the PD-1/PD-L1 pathway is a major inhibitory axis involved in T-cell regulation, peripheral tolerance, and alloimmune responses (8, 41, 51). These analyses are not intended to replace established clinical or histopathological definitions of rejection, but rather to generate mechanistic hypotheses linking exercise responsiveness to immune-metabolic regulation after transplantation.

The long-term follow-up component enhances the study’s clinical relevance. Exercise interventions often demonstrate benefits during supervised periods, but the durability of these effects depends on maintenance of behavior after the structured program ends (52). By classifying participants originally allocated to exercise according to post-intervention exercise continuation or discontinuation, the EXTRA trial can explore whether sustained engagement in exercise is associated with different physiological, biochemical, quality-of-life, and graft-related trajectories. Although this observational phase is subject to confounding, it may provide important information about real-world maintenance of exercise behavior after transplantation.

### 3.1 Strengths and limitations

The trial has several methodological strengths. First, it combines a randomized intervention phase with longitudinal follow-up, allowing the study to examine both short-term treatment effects and longer-term clinical trajectories. Second, the intervention is supervised, progressive, and clinically monitored, which improves internal validity and participant safety. Third, the assessment framework is multidimensional, incorporating cardiopulmonary exercise testing, dual-energy X-ray absorptiometry, handgrip strength, blood pressure, renal and metabolic markers, urinary parameters, inflammatory and immune-checkpoint markers, quality of life, adherence, adverse events, and clinical records for rejection-related outcomes. Fourth, the use of standardized operating procedures and blinded outcome assessment is intended to reduce measurement bias and improve reproducibility.

Several limitations should also be acknowledged. Because of the nature of the intervention, participants and exercise professionals cannot be blinded to group allocation. This is inherent to exercise trials and may influence behavioral or self-reported outcomes, although objective physiological and clinical assessments reduce this concern. The trial may also be underpowered to draw definitive conclusions about rejection, graft survival, or other low-frequency clinical events, particularly if the observed number of events is small. Therefore, graft-related analyses should be interpreted using effect estimates, confidence intervals, event counts, and biological plausibility rather than relying solely on statistical significance. In addition, heterogeneity in donor type, baseline fitness, comorbidity burden, immunosuppressive regimen, and graft function may influence both exercise responsiveness and clinical trajectories. Planned covariate-adjusted and exploratory analyses may help address this variability, but residual confounding may remain, especially in the observational follow-up phase.

Despite these limitations, the EXTRA trial will advance the knowledge on exercise training as a post-transplant intervention that extends beyond traditional rehabilitation endpoints. By integrating various transplant-related outcomes following supervised combined training, the study may help clarify whether the restoration of physiological reserve after transplantation is associated with clinically relevant outcomes. In conclusion, the EXTRA trial evaluates a supervised, progressive, combined aerobic and resistance exercise training program comprising a 2-week familiarization period followed by 24 weeks of structured training. The protocol is designed to determine whether exercise training improves physiological, functional, biochemical, quality-of-life, immune-related, and graft-related outcomes during post-transplant recovery. By bridging exercise science and transplant medicine, this trial may contribute to a more integrated framework for long-term care after kidney transplantation and inform future studies testing exercise as a modifiable strategy to improve transplant survivorship.
