# Supplementary material for: Exercise training in kidney transplant recipients (EXTRA Trial): protocol for a randomized controlled trial
Source: Front Nephrol. 2026 Oct 26;6:1972911. doi: 10.3389/fneph.2026.1972911 (PMC13745256; doi:10.3389/fneph.2026.1972911)
Supplement: Supplementary file 3 [file Table2.docx]

***Supplementary Material 3***

# 1 Detailed assessment procedures and operational definitions

This supplementary material describes the assessment procedures, instruments, and operational definitions used in the EXTRA trial. All assessments are performed by trained personnel using standardized operating procedures. Whenever possible, the same equipment, procedures, and evaluator instructions are maintained across time points to reduce measurement variability. Participants are evaluated at prespecified study visits according to the trial schedule. Clinical and laboratory variables are recorded from direct assessments, biological samples, and transplant follow-up documentation.

## 1.1 Anthropometry

Body weight and height are measured using a calibrated Filizola scale and stadiometer. Participants are assessed barefoot, wearing light clothing, and with pockets emptied. Body weight is recorded in kilograms with the participant standing still in the center of the platform. Standing height is measured with the participant positioned upright on the stadiometer, heels together, body aligned with the vertical board, and head positioned in the Frankfort horizontal plane. The headpiece is lowered to compress the hair and contact the crown of the head. Measurements are recorded at the device's resolution. Body mass index is calculated as body weight in kilograms divided by height in meters squared.

## 1.2 Body composition and bone measures

Body composition is assessed using dual-energy X-ray absorptiometry (Lunar Prodigy DXA system). The DXA assessment is performed according to standardized manufacturer procedures and international densitometry recommendations. Participants are instructed to remove metallic objects and are positioned supine on the scanning table according to the device-specific protocol. Whole-body scans are used to quantify total and regional body composition, including lean mass, fat mass, body fat percentage, and, when available, appendicular lean mass. Skeletal muscle index is calculated as appendicular lean mass divided by height squared when appendicular lean mass is available. Bone-related measures include bone mineral density and T-score when available. Device calibration and quality-control procedures are performed in accordance with the manufacturer’s recommendations and local laboratory procedures. The same DXA device is used across visits whenever possible.

## 1.3 Handgrip strength and muscle function

Maximal handgrip strength is assessed using a Jamar hand dynamometer. Participants are seated with shoulders adducted and neutrally rotated, elbows flexed at approximately 90°, forearms in neutral position, and wrists in a comfortable position. Participants are instructed to squeeze the dynamometer as hard as possible for a brief maximal effort without performing compensatory trunk or shoulder movements. Trials are performed according to a standardized protocol, with rest between attempts. The highest value or the prespecified summary value across valid attempts is recorded in kilogram-force. Low muscle strength and dynapenia are classified according to the criteria prespecified in the main analysis plan. Sarcopenia-related classifications are derived from the combination of muscle strength and DXA-derived muscle mass indicators, consistent with contemporary sarcopenia frameworks.

## 1.4 Cardiorespiratory fitness

Cardiorespiratory fitness is assessed by cardiopulmonary exercise testing on a cycle ergometer. Testing is performed according to standardized clinical exercise testing procedures. Participants are instructed to avoid vigorous exercise before the test and to follow pretest safety instructions regarding food intake, medication use, and clinical symptoms. During the test, gas exchange, ventilation, heart rate, blood pressure, perceived exertion, and clinical signs or symptoms are monitored according to laboratory procedures. Peak oxygen uptake is the highest oxygen uptake achieved during the test and is expressed in mL·kg⁻¹·min⁻¹. Additional ventilatory and cardiovascular variables are recorded when available. Participants take their usual medications on the test day, and the use and dose of beta-blockers and other negative chronotropic drugs are recorded at each test. Because these drugs blunt the heart rate response, attainment of maximal effort is judged from criteria that do not depend on heart rate, such as the peak respiratory exchange ratio and perceived exertion, and these values are reported for each test. Test termination follows clinical safety criteria, participant exhaustion, or investigator judgment.

## 1.5 Blood pressure

Resting systolic and diastolic blood pressure are measured using an oscillometric device. Measurements are performed after the participant has rested quietly in a seated position, with the back supported, feet flat on the floor, and the arm supported at heart level. An appropriately sized cuff is placed on the upper arm. Participants are instructed to avoid talking during measurement. When repeated measurements are obtained, the average of valid readings is used according to the prespecified protocol. Blood pressure is recorded in mmHg.

## 1.6 Blood collection and clinical biochemistry

Venous blood samples are collected by trained personnel using standardized procedures. Blood samples are obtained after an overnight fast whenever required by the planned biochemical analyses, particularly fasting glucose. Samples are processed according to local laboratory procedures, including appropriate tube selection, clotting time when applicable, centrifugation, aliquoting, and storage conditions. Routine biochemical analyses include serum creatinine, fasting blood glucose, lipid profile, C-reactive protein, parathyroid hormone, phosphorus, and vitamin D when available. Laboratory analyses are performed using automated clinical chemistry or immunoassay platforms according to manufacturer instructions and institutional quality-control procedures.

Fasting blood glucose is measured in venous blood and reported in mg/dL. The lipid profile includes low-density lipoprotein cholesterol, high-density lipoprotein cholesterol, and triglycerides, reported in mg/dL. Lipid measurements are performed on a Cobas clinical chemistry platform (Roche Diagnostics) according to the manufacturer’s instructions. C-reactive protein is measured as part of the routine clinical inflammatory profile and reported in mg/L. Parathyroid hormone, phosphorus, and vitamin D are recorded when available as part of the mineral metabolism profile relevant to kidney transplant follow-up.

## 1.7 Renal function and urinary markers

Renal function is assessed using serum creatinine and estimated glomerular filtration rate. Estimated glomerular filtration rate is calculated using the prespecified equation in the main analysis plan (CKD-EPI 2021) and reported as mL/min/1.73 m² or mL/min, depending on the dataset definition. Kidney function categories are derived according to the prespecified chronic kidney disease classification framework.

Urinary markers are assessed using spot urine and 24-hour urine collection. Participants receive standardized instructions for 24-hour urine collection. The collection begins after discarding the first morning void; all urine passed over the next 24 hours is collected, including the first void on the following morning. Participants are instructed to store the container appropriately during the collection period and to report missed samples, spills, or incomplete collection. Total proteinuria is reported in g/day. Incomplete or implausible 24-hour urine collections are identified according to predefined quality-control procedures and clinical judgment.

## 1.8 Immune and inflammatory biomarkers

Inflammatory cytokines and immune checkpoint markers are measured in blood-derived samples using enzyme-linked immunosorbent assays. Biomarkers include interleukin-6, interleukin-2, tumor necrosis factor, programmed cell death protein 1, and programmed death-ligand 1 when available. Samples are processed, aliquoted, and stored according to kit and laboratory requirements to avoid repeated freeze–thaw cycles. ELISA assays are performed according to the manufacturer instructions applicable to each assay. Standard curves, blanks, calibrators, and internal controls are included as specified by the assay. Samples are analyzed in duplicate whenever sample volume allows. Concentrations are calculated from the standard curve and reported in pg/mL. Values below the lower limit of detection or above the upper limit of quantification are handled according to the prespecified statistical analysis plan.

## 1.9 Habitual physical activity

Habitual physical activity is assessed in both groups with the short form of the International Physical Activity Questionnaire (IPAQ), last 7-day recall, in the version validated for Brazilian Portuguese ^1,2^, administered by trained assessors blinded to group allocation. Data are cleaned, truncated, and scored according to the IPAQ Research Committee guidelines. Weekly energy expenditure (MET-min/week) is calculated as the sum of walking (3.3 METs × minutes × days), moderate-intensity activity (4.0 METs × minutes × days), and vigorous-intensity activity (8.0 METs × minutes × days). Participants are classified into the IPAQ low, moderate, or high activity categories and, for analysis, as physically active (≥600 MET-min/week, equivalent to 150 min per week of moderate-intensity activity) or insufficiently active (<600 MET-min/week). Daily sitting time is reported descriptively. MET-min/week is analyzed as a continuous outcome, log-transformed if necessary, and activity status is used as a categorical outcome, as a covariate in sensitivity analyses, and, together with self-reported structured exercise, to classify exercise continuers and discontinuers during the observational phase.

## 1.10 Health-related quality of life

Health-related quality of life is assessed using the 36-Item Short Form Health Survey (SF-36), version 1, in its validated Brazilian Portuguese version^3^, with a 4-week recall period. Questionnaires are administered at prespecified time points by trained personnel or self-completed by participants under standardized instructions. Domain scores are calculated according to the instrument's scoring instructions. The eight domains are physical functioning, role limitations due to physical problems, bodily pain, general health, vitality, social functioning, role limitations due to emotional problems, and mental health; each is transformed to a 0–100 scale, and all eight are included in the statistical analysis, with physical functioning as the prespecified patient-reported measure of physical functioning. Higher scores indicate better health-related quality of life for the corresponding domain unless otherwise specified. Missing questionnaire items are handled according to the instrument's scoring rules and the prespecified statistical analysis plan.

## 1.11 Clinical, transplant-related, and graft-related outcomes

Clinical and transplant-related variables are abstracted from clinical records and transplant follow-up documentation. These include donor type, time after transplantation, immunosuppressive regimen, medication use, comorbidities, adverse clinical events, graft survival, rejection-related events, and months with functioning graft. Rejection-free survival is defined as the time from randomization to the first documented rejection-related event. Rejection-related events are identified from clinical records, biopsy reports, and source documentation, and each episode is classified into one of the categories below.

Biopsy-proven acute rejection: Rejection diagnosed on a kidney allograft biopsy and graded according to the Banff classification in use at the time of the biopsy ^4^. Episodes are classified as T cell-mediated rejection (grade IA or higher), antibody-mediated rejection (active or chronic active), or mixed rejection. Borderline changes are recorded separately and are counted as rejection events only when treated with anti-rejection therapy. Subclinical rejection found on biopsies performed as part of routine care is recorded and reported separately.

An episode in which a biopsy is not performed or is not diagnostic and that meets both of the following prespecified criteria: (a) graft dysfunction, defined as an unexplained increase in serum creatinine of at least 25% above the most recent stable value, confirmed on a repeat measurement, after exclusion of other causes such as urinary obstruction, calcineurin inhibitor toxicity, volume depletion, infection, or transplant renal artery stenosis; and (b) initiation of anti-rejection therapy by the transplant team (pulse corticosteroids, lymphocyte-depleting antibodies, intravenous immunoglobulin, and/or plasmapheresis). Empirically treated episodes in which a subsequent biopsy excludes rejection are not counted as rejection.

Rejection-free survival is defined as the time from randomization to the first biopsy-proven acute rejection or clinically diagnosed rejection, whichever occurs first; biopsy-proven acute rejection alone is analyzed as a secondary definition. The event date is the date of the diagnostic biopsy or, for clinically diagnosed rejection, the date on which anti-rejection therapy is started. Each episode is reviewed independently by two investigators with transplantation expertise who are unaware of group allocation, and disagreements are resolved by a third investigator.

Graft failure is defined as the first of the following: return to maintenance dialysis, re-transplantation, or transplant nephrectomy. Temporary dialysis for acute kidney injury followed by recovery of graft function is not considered graft failure. The date of graft failure is the date of the first session of maintenance dialysis, the date of re-transplantation, or the date of nephrectomy, whichever occurs first. Death with a functioning graft is recorded as a separate outcome. Death-censored graft survival is the main graft outcome, with death with a functioning graft treated as a competing event; overall graft survival (graft failure or death from any cause, whichever occurs first) is also reported. Months with a functioning graft are counted from transplantation to graft failure, death, or last follow-up.

## 1.12 Medication and comorbidity data

Medication use is recorded from clinical records and participant documentation when available. Immunosuppressive and transplant-related medications include tacrolimus, cyclosporine, mycophenolate mofetil, prednisone, azathioprine, everolimus, sirolimus, cotrimoxazole prophylaxis, and valganciclovir when applicable. Additional medication classes include antihypertensive agents, statins, insulin or oral hypoglycemic agents, diuretics, and vitamin D supplementation. Medication data are treated as binary or categorical exposure variables, depending on the main statistical analysis plan. Comorbidities, including diabetes mellitus and hypertension, are recorded from clinical records.

## 1.13 Adherence, perceived exertion, and safety

Exercise adherence is assessed using attendance records, session logs, and participant exercise diaries. Adherence is calculated as the number of completed sessions divided by the number of prescribed sessions, multiplied by 100, over the 24-week intervention, excluding the familiarization period (72 resistance and 68 aerobic sessions; 140 sessions in total). It is reported for the whole program and separately for each training component. Missed sessions count as not completed, and the reason is recorded; sessions postponed or suspended for clinical reasons remain in the denominator. Partially completed sessions are recorded with the proportion of the prescribed volume that was performed (sets and repetitions for resistance exercise; minutes for walking), and that proportion is added to the numerator. Participants who complete at least 70% of the prescribed sessions (at least 98 of 140) are classified as adherent, and this threshold defines the per-protocol population. It was chosen to accommodate the intercurrent clinical events expected during the first post-transplant year while still ensuring substantial exposure to training. Perceived exertion is monitored using the Brazilian Portugese version of Borg Rating of Perceived Exertion scale (6–20 version, from 6, no exertion, to 20, maximal exertion) ^5^ during the intervention period. Safety monitoring includes participant-reported symptoms, adverse events, clinical events documented in medical records, and investigator assessment during supervised sessions. Participants are instructed to stop exercise and contact the study team if they experience chest pain, severe dyspnea, dizziness, palpitations, unusual fatigue, or any other concerning symptom. Pre-session criteria for postponing a session, criteria for stopping exercise, and criteria for suspending or discontinuing the intervention are described in the main manuscript and were adapted from the American Heart Association exercise standards ^6^.

An adverse event is any untoward medical occurrence in a participant during the study, whether or not it is considered related to the intervention. A serious adverse event is any adverse event that results in death, is life-threatening, requires inpatient hospitalization or prolongation of an existing hospitalization, results in persistent or significant disability or incapacity, or is considered an important medical event that may require intervention to prevent one of these outcomes (8). Events anticipated with exercise training include delayed-onset muscle soreness, musculoskeletal injury, falls, exercise-related hypotension or hypoglycemia, and cardiovascular symptoms during exercise.

Adverse events are ascertained actively at every supervised session in the exercise group and at every assessment visit and scheduled follow-up contact in both groups, using the same open question about new symptoms, illnesses, injuries, emergency visits, and hospital admissions, and are complemented by review of medical records. For each event, the date, description, severity, seriousness, relationship to exercise, action taken, and outcome are recorded. When an applicable term is available severity is graded with the Common Terminology Criteria for Adverse Events, version 5.0 (grade 1, mild; grade 2, moderate; grade 3, severe or medically significant; grade 4, life-threatening; grade 5, death). Relationship to exercise is classified by the investigator as unrelated, unlikely, possibly, probably, or definitely related. Serious adverse events are reported to the principal investigator within 24 hours and notified to the research ethics committee in accordance with national regulations.

Adverse events and serious adverse events are reported by group and study phase as the number of events and the number and proportion of participants with at least one event, following the CONSORT Harms 2022 statement ^7^. Between-group differences are expressed as risk differences with 95% confidence intervals and tested with Fisher's exact test; incidence rates per 100 participant-months are also reported. Events related to exercise are summarized separately.

## 1.14 References

1	Craig, C. L. *et al.* International physical activity questionnaire: 12-country reliability and validity. *Med Sci Sports Exerc* **35**, 1381-1395, doi:10.1249/01.MSS.0000078924.61453.FB (2003).

2	Matsudo S, A. T., Matsudo V, Andrade D, Andrade E, Oliveira LC, et al. QUESTIONÁRIO INTERNACIONAL DE ATIVIDADE FÍSICA (IPAQ): ESTUDO DE VALIDADE E REPRODUTIBILIDADE NO BRASIL. *Rev. Bras. Ativ. Fís. Saúde* **6**, 5-18, doi:10.12820/rbafs.v.6n2p5-18 (2001).

3	Ciconelli, R. M., Ferraz, M.B., Santos, W., Meinão, I. and Quaresma, M.R. Brazilian-Portuguese version of the SF-36. A reliable and valid quality of life outcome measure *Rev bras reumatol* **39**, 143–150 (1999).

4	Naesens, M. *et al.* The Banff 2024 Kidney Meeting Report: Rejection as a spectrum of phenotypes and focus on differential diagnostic reasoning. *Am J Transplant* **26**, 922-936, doi:10.1016/j.ajt.2026.01.018 (2026).

5	Cabral, L. L. *et al.* Initial Validity and Reliability of the Portuguese Borg Rating of Perceived Exertion 6-20 Scale. *Measurement in Physical Education and Exercise Science* **24**, 103-114, doi:10.1080/1091367x.2019.1710709 (2020).

6	Fletcher, G. F. *et al.* Exercise standards for testing and training: a scientific statement from the American Heart Association. *Circulation* **128**, 873-934, doi:10.1161/CIR.0b013e31829b5b44 (2013).

7	Junqueira, D. R. *et al.* CONSORT Harms 2022 statement, explanation, and elaboration: updated guideline for the reporting of harms in randomised trials. *BMJ* **381**, e073725, doi:10.1136/bmj-2022-073725 (2023).
